# Antifungal Activity and Action Mechanism of Histatin 5-Halocidin Hybrid Peptides against *Candida ssp*

**DOI:** 10.1371/journal.pone.0150196

**Published:** 2016-02-26

**Authors:** Juhye Han, Md. Anirban Jyoti, Ho-Yeon Song, Woong Sik Jang

**Affiliations:** 1 Department of Microbiology and Immunology, School of Medicine, Soonchunhyang University, Cheonan, Chungnam, 31151, South Korea; 2 Regional Innovation Center, Soonchunhyang University, Asan, Chungnam, 31538, South Korea; Bose Institute, INDIA

## Abstract

The candidacidal activity of histatin 5 is initiated through cell wall binding, followed by translocation and intracellular targeting, while the halocidin peptide exerts its activity by attacking the Candida cell membrane. To improve antimicrobial activities and to understand the killing mechanism of two peptides, six hybrid peptides were designed by conjugating histatin 5 and halocidin. A comparative approach was established to study the activity, salt tolerance, cell wall glucan binding assay, cytotoxicity, generation of ROS and killing kinetics. CD spectrometry was conducted to evaluate secondary structures of these hybrid peptides. Furthermore the cellular localization of hybrid peptides was investigated by confocal fluorescence microscopy. Of the six hybrid congeners, di-PH2, di-WP2 and HHP1 had stronger activities than other hybrid peptides against all tested Candida strains. The MIC values of these peptides were 1–2, 2–4 and 2–4 μg/ml, respectively. Moreover, none of the hybrid peptides was cytotoxic in the hemolytic assay and cell-based cytotoxicity assay. Confocal laser microscopy showed that di-PH2 and HHP1 were translocated into cytoplasm whereas di-WP2 was accumulated on surface of *C*. *albicans* to exert their candidacidal activity. All translocated peptides (Hst 5, P113, di-PH2) were capable of generating intracellular ROS except HHP1. Additionally, the KFH residues at C-terminal end of these peptides were assumed for core sequence for active translocation.

## 1.0 Introduction

Antimicrobial peptides (AMP) are recognized as one of the primordial and evolutionary defense systems of the animal kingdom. AMPs are widely distributed in nature and therefore play pivotal roles in the innate immune system of many organisms [1–3]. AMPs, found in every tissue system also protect human beings continually against pathogenic invasion and diseases from viruses, bacteria, fungi, protozoa and other parasites, thus retaining their therapeutic potential as ‘peptide-antibiotic’ in humans as well [3, 4]. Moreover, AMPs for antibiotic applications are considered advantageous over conventional antibiotics as conventional antibiotics contribute to the emergence of resistant pathogens and fail to treat diseases from resistant pathogens afterward [5]. The therapeutic potential of a majority of AMPs are due to their ability to destabilize the cytoplasmic membrane of pathogens and the ability to kill multi-drug resistant pathogens as well as rapid killing kinetics.

Histatin 5 (Hst 5), one of the salivary histatins, is amphiphatic with α helices and inhibits fungus and bacteria. Of the three major human histatins (Hst 1, Hst 3 and Hst 5), Hst 5 is the most potent anticandidal peptide and kills both blastopore and germinated forms of *Candida albicans* [6]. Hst 5, a 24 amino acid residual peptide, is produced with multiple posttranslational proteolytic processing of Hst 3. A number of studies on the killing mechanism of Hst 5 showed that rather than a single target, multiple intracellular targets are orchestrated, leading to osmotic imbalance via the loss of potassium ions and triggering candidacidal activity [7, 8]. In addition, Helmerhorst *et al*. reported that histatin 5 induced the formation of reactive oxygen species (ROS) in *C*. *albicans* cells as well as in isolated mitochondria and that ROS levels were highly correlated with cell death [9]. The cellular uptake of Hst 5 involves one or all of the three uptake pathways: i) transporter mediated uptake, ii) direct transfer across the membrane and iii) receptor mediated endocytosis [10]. Also, it was shown that the structural conformation of Hst 5 is important for membrane binding and killing of *C*. *albicans*. At least 12 amino acids at the C terminus of Hst 5 form the functional domain required for candidacidal activity [11]. Based on the full length sequence of Hst 5, synthetic congeners of Hst 5 have been synthesized and investigated for more biological activities exceeding that of the parental Hst 5 [12]. One modified congener of Hst 5, P113, contains 12 amino acid residues from Hst 5 and was found to retain the anticandida activities. The mode of action for P113 was assumed to be similar to that of Hst 5 [13].

Halocidin is a heterodimeric peptide from tunicate *Halocynthia aurantium* with α-helical structure and is reported to exert a broad spectrum of antimicrobial activities in vitro [14, 15]. The heterodimer comprises two different subunits; one contains a total of 18 amino acid residues (WLNALLHHGLNCAKGVLA, 18Hc) and the other contains only 15 (ALLHHGLNCAKGVLA, 15Hc). Both subunits are linked covalently by an inter-disulfide bond. Previous studies reported that the dimeric congeners of halocidin are stronger antimicrobials than their monomeric forms [14, 15]. However, the target for candidacidal activity differs from that of Hst 5 and P113 because halocidin disrupts the cell membrane rather than intracellular translocating [15]. Interestingly, halocidin was found to retain its candidacidal activity under physiological salt conditions [15].

To design an antibiotic of peptide origin, several limitations have to be overcome. Conformational analysis and residual amino acid rearrangement of AMPs suggest that synthetic peptides with higher activity, high salt tolerance and low cytotoxicity can be achieved. Both P113 and halocidin are potentially active against fungi but differ in their conformations and mode of action. P113 is a monomeric peptide with very weak α-helices in the secondary structure, whereas halocidin is a dimeric peptide with moderate α-helices. As stated elsewhere, both Hst 5 and P113 lose their candidacidal activities in physiologic salt environments [11]. In contrast, halocidin is relatively resistant to elevated concentrations of NaCl and MgCl_2_ but one disadvantage of halocidin can be attributed to its slightly hemolytic activity [14]. In halocidin structure, its large subunit (18Hc) differs from the small one (15Hc) only by containing three additional N-terminal amino acid residues (Trp-Leu-Asn) [13]. However, 15Hc showed much less antimicrobial activity than 18Hc with an extra N-terminal part. Thus, it was reported that N-terminal three amino acid residues (WLN) of 18Hc is important for the antimicrobial activity of this peptide [13]. Interestingly, N-terminal six amino acid residues (ALLHHG) of 15Hc are somewhat homologous to that (AKRHHG) of P113. Therefore, in this study, hybrid congeners were synthesized by adding the N-terminal three amino acid sequences (WLN) of 18Hc to P113 or conjugating the amino acid sequences of P113 and 18Hc so that the aforementioned shortcomings of these peptides could be circumvented. We here assessed the anti-candida activities, the killing kinetics, ROS formation and cytotoxicity of these hybrid peptide congeners. We also investigated the target sites of these hybrid congeners to determine whether they could be translocated to cytosol or localized into membranes to destabilize *Candida albicans*.

## 2.0 Materials and Methods

### 2.1 Candida Strains and Materials

To determine the candidacidal activity of the peptides, we used Candida strains obtained from the Culture collection of antimicrobial resistant microbes at Seoul Women’s University in Korea. These included three *Candida albicans* (CCARM 14020, 14021, 14022), *Candida guillermondi* and *Candida tropicalis*. In addition, *Candida albicans* SC5413 was kindly provided by Dr Jeong-Yoon Kim at the Department of Microbiology & Molecular Biology, Chungnam University, Korea. Yeast were cultured overnight at 30°C in 10 ml of yeast extract-peptone-dextrose (YPD) broth (Qbiogene), then diluted in 10 ml of YPD broth to OD_600_ = 0.2 and grown for an additional 4 h with shaking at 30°C until OD_600_ = 0.8 in order to obtain mid-log phase cultures. The peptides (Hst 5, P113, HHP1, HHP2, PH1, PH2, di-PH1, di-PH2, WP1, di-WP2, di-15Hc and di-18Hc) in this study were synthesized by using standard solid-phase synthesis protocols and were purified by reversed-phase high-performance liquid chromatography at Anygen Inc. (Korea). FITC-labeled peptides (F-Hst5, F-P113, F-HHP1, F-di-PH2, F-di-WP2 and F-di-18Hc) were also synthesized by 6-aminohexnoic acid linker at Anygen (Korea) for confocal experiments. The primary structures of these peptides are shown in Table 1.

**Table 1:** Amino acid sequence of histatin 5, halocidin and hybrid peptides.

Peptide	Amino acid sequence																									
Hst 5	D	S	H	A	K	R	H	H	G	Y	K	R	K	F	H	E	K	H		H	S	H	R	G	Y	^a^
P113				A	K	R	H	H	G	Y	K	R	K	F	H	^a^										
PH1				A	K	R	H	H	G	L	N	C	A	K	G	V	L	A	^a^							
PH2				A	K	R	H	H	G	L	N	C	A	K	F	H	^a^									
HHP2				A	L	L	H	H	G	Y	K	R	K	F	H	^a^										
HHP1	W	L	N	A	L	L	H	H	G	Y	K	R	K	F	H	^a^										
WP1	W	L	N	A	K	R	H	H	G	Y	K	R	K	F	H	^a^										
WP2	W	L	N	A	K	R	H	H	G	Y	K	C	K	F	H	^a^										
15Hc				A	L	L	H	H	G	L	N	C	A	K	G	V	L	A	^a^							
18Hc	W	L	N	A	L	L	H	H	G	L	N	C	A	K	G	V	L	A	^a^							

^a^ Asterisks signify C-terminal amidation

### 2.2 Candidacidal Assay

The candidacidal activities of the peptides were tested by using standard microdilution plate assays [16], radial diffusion assays [17] and broth dilution assay slightly modified from the procedure recommended by the National Committee for Clinical Laboratory Standards [14]. For the candidacidal assays, *C*. *albicans* cells were grown in YPD medium and washed twice with 10 mM sodium phosphate buffer (NaPB; Na_2_HPO_4_, NaH_2_PO_4_, pH 7.4). The cells (1 x 10^6^) were then mixed with different concentrations of peptides and incubated at 30°C with constant shaking for 60 min. For energy depletion experiments, cells were pre-incubated with 10 mM NaPB containing 5 mM NaN_3_ at 30°C for 60 min before the addition of peptides. The cell suspensions were diluted in 10 mM NaPB and aliquots of 500 cells were spread onto YPD agar plates then incubated for 24 h at 30°C to visualize the surviving colonies. Cell survival was expressed as a percentage of that for the control, and the loss of viability was calculated as [1 x (number of colonies from peptide-treated cells/number of colonies from control cells)] x 100. Candidacidal assays were performed in triplicate. For the radial diffusion assay, *C*. *albicans* cells were grown in YPD medium overnight and washed twice with 10 mM NaPB. Washed yeast-phase *C*. *albicans* cells (1 x 10^6^ CFU) were trapped in thin underlay gels containing 9 mM sodium phosphate, 1 mM sodium citrate buffer, 1% (wt/vol) agarose (A6013; Sigma), and 0.3 mg of Sabouraud dextrose broth (Difco)/ml. After incubation at 37°C for 3 h, a 10 ml overlay gel of 1% agarose and 6% Sabouraud dextrose broth was poured on the underlay gel and then the plates were incubated overnight at 37°C. The clear-zone diameters were measured to the nearest 0.1 mm and graphed against the peptide concentration. Zone diameters were expressed in units (0.1 mm = 1 unit). For the broth dilution assay, the Candida strains were cultured in LYM broth [18] for 18 h at 30°C. The numbers of yeasts were counted using a hemocytometer. Stock peptide solutions were prepared to a concentration of 320 μg/ml in distilled water, and were then serially diluted twofold, to 5 μg/ml. 100 μl aliquots of the diluted culture media containing candida cells (1 x 10^3^) were dispensed into the wells of 96-well polypropylene microtiter plates (Costar 3879, Corning, NY), to which peptide solution (11 μl) was then added. In the experiments conducted to assess the candidacidal activity of peptides in the presence of salt, we used LYM supplemented with 150 mM NaCl for the culture medium, which was mixed with the peptide solution. The antifungal activities were evaluated after 24 h of incubation at 30°C by the observation of visible turbidity the wells. The MIC values were expressed as intervals (a–b), with a representing the highest tested concentration at which microbes continue to grow, and b representing the lowest concentration at which growth inhibition could be detected.

### 2.3 Propidium Iodide Assay

To confirm the killing effect of the peptides, washed Candida cells (1 × 10^7^ cells/ml) were treated with the peptides and propidium iodide (PI) was added. For energy depletion experiments, cells were pre-incubated with 10 mM NaPB containing 5 mM NaN_3_ at 30°C for 60 min before the addition of peptides. Propidium iodide (PI) uptake was measured using a multi reader and calculated with the formula [propidium iodide (PI) uptake value of peptide-treated cells—propidium iodide (PI) value of control cells]

### 2.4 Hemolysis Assay

20 μl of peptides at pre-determined concentrations were added to 180 μl of a 2.5% (V/V) suspension of rabbit erythrocytes in phosphate-buffered saline (PBS). Melittin (Sigma), a hemolytic peptide from bee venom, was used as the positive control sample. The mixture was incubated in 96-well polypropylene microtiter plates for 30 min at 37°C. After centrifugation at 3,000 rpm for 10 min, 100 μl of the supernatant was added to new 96-well plates and the absorbance measured at 540 nm by using a Perkin Elmer 2030 Reader (Victor X3).

### 2.5 Cytotoxic Activity of Hybrid Peptides

The cytotoxicity of hybrid peptides was evaluated in mouse epithelial cells (L929, Korean Collection for Type Cultures, KCTC). L929 cells in RPMI1640-10% FBS (100 μL, 3 × 10^5^ cells/ml) were seeded in 96-well plates and cultured at 37°C for 24 h in a 5% CO_2_ incubator. The L929 cells were treated with peptides at predetermined concentrations for 24 h, and 10 μl of EZ-Cytox Cell Viability Assay solution WST-1^®^ (Daeil Lab Service, Korea) were added to each well. Three hours later, the cytotoxicity of peptides against L929 cells was assessed by measuring the optical density at 460 nm using a Perkin Elmer 2030 Reader (Victor X3).

### 2.6 Binding Assay for Binding of the Peptides to Candida Cell Wall Component

Binding of hybrid peptides to the surface of *C*. *albicans* cells was examined by assessing the inhibitory effect of fungal cell wall components on the antimicrobial activity of the hybrid peptides. 20 μl of a pre-determined concentration of the peptides was added to 80 μl of each polysaccharide (0.07–40 mg/ml in 10 mM NaPB) including laminarin (β-1,3-glucan polymer; Sigma), pustulan (β-1,6-glucan polymer; Calbiochem) or mannan (mannose polymer; Sigma) and incubated for 2 h at 37°C. The inhibitory effects of the polysaccharides on the antimicrobial activity of the peptides were assessed by radial diffusion assay as described above.

### 2.7 CD Spectra

Circular dichroism (CD) spectra of the six peptides (Hst5, P113, HHP1, di-PH2, di-WP2 and di-18Hc) were measured with a J-715 spectropolarimeter (Jasco, Tokyo, Japan). Two hundred micrograms of each peptide was resuspended in 1 ml of 10 mM NaPB (pH 7.0) (i) without other additives, (ii) with 10% laminarin (LAM) or (iii) with 50% trifluoroethanol (TFE) (a known helix-inducing solvent). The spectra were measured at room temperature in a 1-mm-path-length quartz cell. The scanning speed was 10 nm/min (180 to 260 nm), and each spectrum was the average of five scans.

### 2.8 Confocal Microscopy

*C*. *albicans* cells were attached to concanavalin A (Sigma, 1 mg/ml solution in water) coated coverglass in chambered wells (Lab-Tek II). Cells (1 x 10^6^) were deposited in each well, and 1 ml of 10 mM NaPB containing 5 μg PI (Sigma) and FITC-labeled peptides (Final concentration 10 μg/ml) were added to the chamber. Confocal images were acquired with an Olympus Fluoview FV10i-DOC (Olympus, Tokyo, Japan). ImageJ software was used for image acquisition and analysis.

### 2.9 Reactive Oxygen Species (ROS) Production

The amount of ROS produced by Candida cells treated with peptides was measured by a fluorometric assay with DCFHDA as described earlier [19]. Briefly, the cells were adjusted to an OD_600_ of 1 in 10 ml of NaPB and centrifuged at 5000 ×g for 10 min. The cell pellet was then resuspended in NaPB and treated with peptides alone or with ascorbic acid. After the addition of 10 μM 2`,7`dichlorofluorescein diacetate (DCFH-DA), the fluorescence intensity of the induced ROS production was recorded every 10 min at excitation and emission wavelengths of 485 nm and 530 nm, respectively, in a Perkin Elmer 2030 Reader (Victor X3).

## 3.0 Results

### 3.1 Candidacidal Activity of Hst 5, P113, di-18Hc and Hybrid Peptides

We designed 6 hybrid peptides based on the primary structures of P113 and 18Hc (Table 1). PH1 was designed by connecting the N-terminal part of P113 (1–6^st^ amino acid) to the C-terminal part of 18Hc (10–18^st^ amino acid). PH2 was synthesized by the substitution of four C-terminal amino acid residues (GVLA) in PH1 with F-H residues. HHP1 was designed with the N-terminal part of 18Hc (1–8^st^ amino acid) and the C-terminal part of P113 (6–12^st^ amino acid) while HHP2 was synthesized by the subtraction of three N-terminal amino acid (WLN) residues from HHP1. WP1 was designed using the N-terminal part of 18Hc (1–3^st^ amino acid) and the C-terminal part of P113 (1–12^st^ amino acid). WP2 was synthesized by substitution of the R residue (12^st^ amino acid) in WP1 with a Cysteine residue. All peptides with cysteine residues (PH1, PH2 and WP2) were also converted into the homodimeric version. Table 2 shows the anti-Candida activities (MICs) of hybrid peptides and control peptides (Hst 5, P113 and di-18Hc) against four *Candida albicans* strains. The anti-Candida activities of PH1, di-PH1, HHP2 and WP1 were reduced almost entirely, whereas HHP1, di-PH2 and di-WP2 had stronger activities than other hybrid peptides against all tested Candida strains. Comparing the dimeric and monomeric forms of three hybrid peptides (PH1, PH2 and WP2), the dimeric form had stronger candidacidal activities than the corresponding monomer. The highest antifungal activity against all of the strains of *C*. *albicans* was found in the hybrid dimer di-PH2.

**Table 2:** MICs of Hst 5, P113, di-18Hc and hybrid peptides against Candida strains.

	MICs (μg/ml)			
Peptide	*C*.*albicans* SC5413	*C*.*albicans* CCARM 14020	*C*.*albicans* CCARM 14021	*C*.*albicans* CCARM 14022
Hst 5	8–16	8–16	4–8	8–16
P113	4–8	4–8	4–8	8–16
**HHP1**	**2–4**	**2–4**	**2–4**	**4–8**
HHP2	16–32	8–16	32<	32<
PH1	16–32	16–32	8–16	8–16
PH2	1–2	1–2	1–2	8–16
di-PH1	16–32	16–32	4–8	4–8
**di-PH2**	**1–2**	**1–2**	**1–2**	**2–4**
WP1	4–8	4–8	8–16	32<
**di-WP2**	**2–4**	**4–8**	**4–8**	**4–8**
di-18Hc	2–4	2–4	2–4	2–4

### 3.2 Hemolysis Activity and Cytotoxicity of Hybrid Peptides

**Fig 1:**
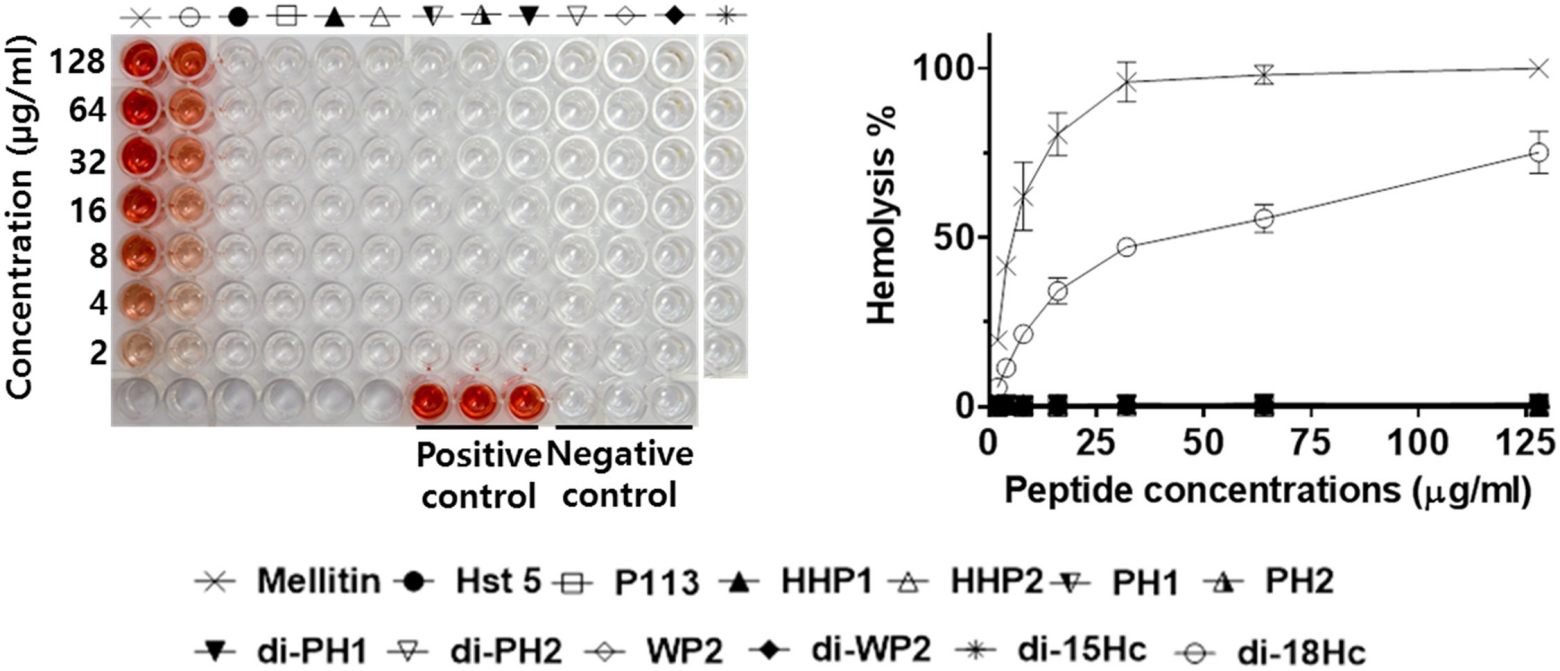
Hemolytic activities of Hst5, P113, di-18Hc, Halocidin-P113 hybrid peptides and control peptide. 1% of triton-X100 was used as the control for 100% hemolysis and 0.01% acetic acid was used as the peptide-free control. Percent hemolysis was calculated with the following equation: Hemolysis (%) = (A_540_ of sample—A_540_ of peptide-free control) / (A_540_ of 100% control—A_540_ of peptide-free control) × 100.

One of the prerequisite for antibiotic drug development is no lethal activity by the drug on human cells or that it is at least kept within a minimum range free of harmful side effects over the course of treatment [20]. So far, antimicrobial peptides are thought to be free of cytotoxicity due to such a non-specific membrane-lytic mechanism [21]. However, not all peptides are in this class. Detrimental cytotoxicity of AMPs has been reported [22]. For example, the cytotoxicity of melittin on different mammalian cells including erythrocytes is well known [23]. In this study, we therefore included melittin as a control in the hemolysis activity assay and compared the results with Hst 5, P113, halocidin and our hybrid congeners (Fig 1). As reported in a previous papers [24, 25], our hemolysis assay also showed that Hst5 and P113 had no hemolysis activity at 128 μg/ml. In contrast, halocidin (di-18Hc) showed hemolytic activity of 75% at 128 μg/ml. In a previous study, halocidin was reported to be moderately cytotoxic due to having a cytotoxic monomer 18Hc [26]. Interestingly, all hybrid peptides lacked hemolysis activity up to 128 μg/ml. In addition, the cytotoxic activities of three hybrid peptides (HHP1, di-pH2 and di-WP2) and three control peptides (Hst 5, P113 and di-18Hc) were determined in L929 cells. An MTT assay showed that the cytotoxicity of all tested peptides was lower than their antimicrobial activity (Table 3). CC_50_ values of HHP1, di-WP2, di-PH2 and two control peptides (Hst 5 and P113) were over 200 μg/ml, while di-18Hc eliminated 50% of L929 cells at 72.385 μg/ml.

**Table 3:** CC_50_ values of Hst 5, P113, di-18Hc and hybrid peptides against L929 cells.

	CC_50_ (μg/ml)
Peptide	L929 cells
Hst 5	200<
P113	200<
HHP1	200<
di-PH2	200<
di-WP2	200<
di-18Hc	72.385

### 3.3 Candidacidal Activities of Peptides in the Presence of Salts

To assess the inhibitory effects of salt on the candidacidal activities of the peptides, Hst 5, P113, di-18Hc and three hybrid peptides (HHP1, di-PH2 and di-WP2) were tested for candidacidal activities in 150 mM NaCl against four *C*. *albicans* strains and two non-albicans strains (*C*. *guilliermondi* and *C*. *tropicalis*). The MICs of peptides in 150 mM NaCl concentrations against Candida strains are shown in Table 4. The killing activities of Hst 5, P113, HHP1 and di-PH2 against *C*. *albicans* strains were abolished in 150 mM salt. In contrast, the killing activities of di-WP2 and di-18Hc were 4-8-fold decreased but not abolished in 150 mM salt. Interestingly, two hybrid peptides (di-WP2 and di-PH2) and di-18Hc retained its killing activities against *C*. *tropicalis* in 150 mM NaCl salt. di-WP2 retained its activity against *C*. *guilliermondi* and *C*. *tropicalis* over 150 mM salt condition, whereas di-PH2 showed a 2-fold increase in MIC against *C*. *tropicalis* but no activity against *C*. *guilliermondi* at 150 mM salt (MICs: > 32 μg/ml). It should be noted here that the non-albicans were found susceptible to dimeric peptides of di-PH2, di-WP2 and di-18Hc even at 150 mM salt concentrations with only exception of di-PH2 against *C*. *guilliermondi*.

**Table 4:** Effects of salt on MICs of Hst 5, P113, di-18Hc and hybrid peptides against Candida strains.

	MICs (μg/ml)					
NaCl Conc (mM)	*C*. *albicans* SC5413	*C*. *albicans* CCARM14020	*C*. *albicans* CCARM 14021	*C*. *albicans* CCARM 14022	*C*. *guilliermondi* CCARM 14018	*C*. *guilliermondi* CCARM 14019
**Hst 5**						
**0**	8–16	8–16	4–8	8–16	1–2	2–4
**150**	32<	32<	32<	32<	32<	32<
**P113**						
**0**	4–8	4–8	4–8	8–16	0.5–1	0.5–1
**150**	32<	32<	32<	32<	32<	32<
**HHP1**						
**0**	2–4	2–4	2–4	4–8	1–2	1–2
**150**	32<	32<	32<	32<	32<	8–16
**di-PH2**						
**0**	1–2	1–2	1–2	2–4	**0.5–1**	**0.5–1**
**150**	32<	32<	32<	32<	**32<**	**1–2**
**di-WP2**						
**0**	2–4	4–8	4–8	4–8	**2–4**	**4–8**
**150**	8–16	16–32	8–16	16–32	**2–4**	**4–8**
**di-18Hc**						
**0**	2–4	2–4	2–4	2–4	2–4	4–8
**150**	16–32	16–32	16–32	16–32	4–8	4–8

### 3.4 Binding of Hybrid Peptides to the Cell Surface of *C*. *albicans* via a Specific Interaction with β-1,3-Glucan

**Fig 2:**
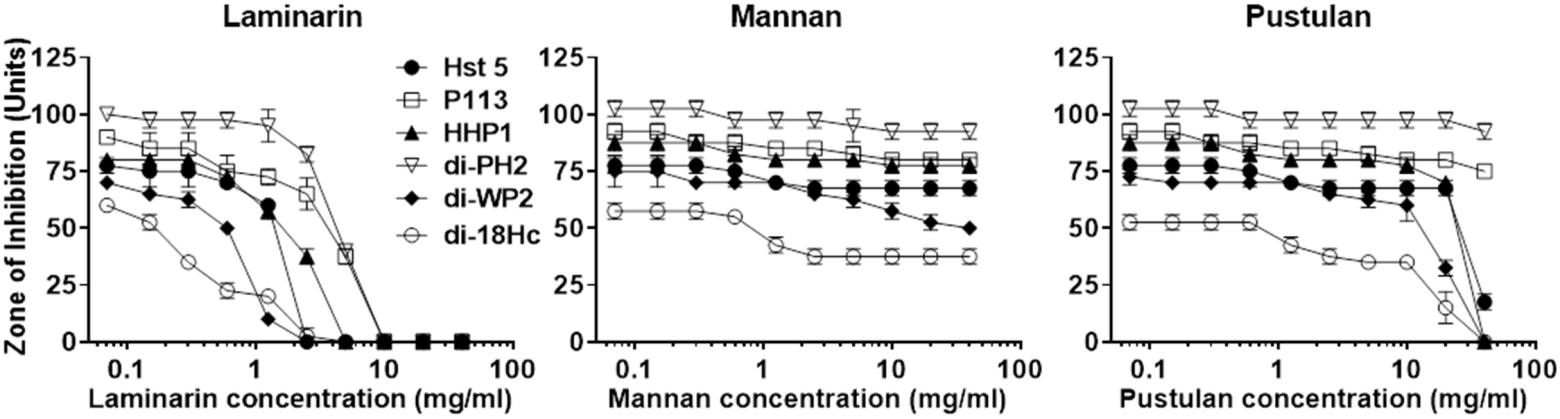
Specific binding of Hst 5, P113, hybrid peptides and di-18Hc to laminarin. Binding/radial diffusion assay was performed by mixing various amounts of Candida cell wall components (laminarin, mannan and pustulan) with test peptides (final concentration of 180 μg/ml). 5 μl of each mixed samples were loaded into the well in the agar plate and 5 μl of each peptide solutions (180 μg/ml) was used for the polysaccharides-free control. The anti-Candida activities of peptides in the mixture were graphed against the concentration of polysaccharides. Diameters of the clearing zone are expressed in units (1 mm = 10 U).

Initial binding of Hst 5 and di-18Hc to cell wall β-1,3-glucan is important for exerting candidacidal activity [10, 15]. In this study, we investigated the capabilities of our hybrid peptides for binding specifically to β-1,3-glucan via assessing the inhibitory effects of β-1,3-glucan on the anti-Candida activity. After a constant amount of hybrid peptides were pre-incubated with different concentrations of laminarin (β-1,3-glucan), the mixtures were tested for anti-Candida activity in radial diffusion assays. The antimicrobial activity of Hst 5, P113, HHP1, di-PH2, di-WP2 and di-18Hc was drastically decreased by pre-incubation with laminarin at concentration of 2.5, 5, 2.5, 5, 1.25 and 0.3 mg/ml, respectively (Fig 2), whereas mannan did not affect the anti-Candida activity of the hybrid peptides. In case of pustulan (β-1,6-glucan), the activity of HHP1, di-WP2 and di-18Hc began to decrease at 20 mg/ml of pustulan. The activities of all hybrid peptides were reduced in proportion with the loss of cell wall binding as the amount of laminarin in the mixture increased, similar to the results from the pre-incubation of Hst 5 or di-18Hc with laminarin.

### 3.5 CD Spectrometry

**Fig 3:**
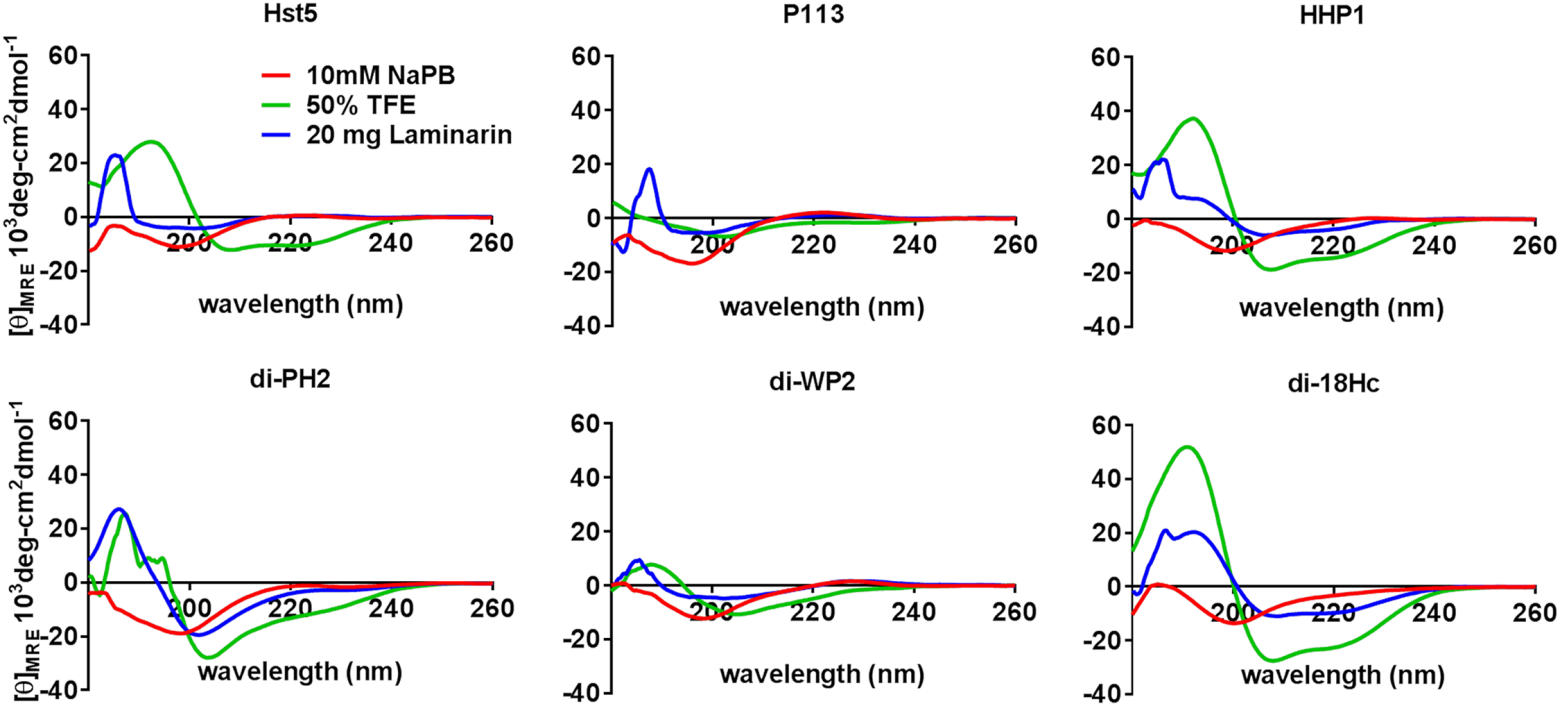
CD spectra of Hst5, P113, di-18Hc and hybrid peptides. This study was performed with one concentration (200 μg/ml) of each peptide in various buffers: 10 mM sodium phosphate buffer (Red line), 50% TFE in phosphate buffer (green line) and 20 mg of laminarin in phosphate buffer.

CD-spectrometry analysis narrates the secondary structure of an AMP in a cell physiologic mimicry condition by determining the level of α-helicity. It was reported that Hst 5 and di-18Hc spectra obtained in the presence of TFE has the typical appearance of α-helix-rich structures [14, 27], whereas P113 showed fewer features characteristic of an α helix [13]. To determine whether hybrid peptides retained the ability to form an α helix in membrane-mimicking environments, the CD spectra of the six peptides were measured in 10 mM NaP buffer with 50% TFE (Fig 3) and analyzed to determine their secondary structures using spectra manager software. In the presence of TFE, HHP1 spectra had the appearance of α-helix-rich structures with dichroic minimal values at 208 and 222 nm and a maximum near 194 nm. However, di-PH2 and di-WP2 showed fewer features characteristic of an α helix. According to the results of secondary structure analysis with spectra manager software program (Table 5), the α-helix content of HHP1 in a hydrophobic environment was 36.8% while those of di-PH2 and di-WP2 were 10.1% and 7.8%, respectively. The α-helix contents of Hst 5, di-18Hc and P113 in the presence of TFE were 76.2%, 47% and 4.4% respectively. The spectra for all tested peptides in 10 mM NaP buffer were characteristic of unordered structures. Next, we tested the spectra of peptides in 20 mg/ml of laminarin in the buffer to conform the secondary structures of the peptides in the presence of β-1,3-glucan, which is the initial binding component in the Candida cell wall for all tested peptides. The α-helix content of di-PH2 and di-18Hc with laminarin in the buffer was 20.5% and 29.2%, respectively. The α-helix contents for the other peptides were below 11%. According to CD spectrometric analysis, the highest α-helices were formed in the order of Hst 5>di-18Hc>HHP>di-PH2>Di-WP2>P113 in 50% TFE. Interestingly, both di-18Hc and di-PH2 also exhibited a moderate amount of helical folding in laminarin. It is generally agreed that α-helical secondary peptides can interact with cytoplasmic membranes or cell surface. However, the higher degree of α-helix orientation does not guarantee higher antimicrobial activity by the hybrid peptides as observed in this study.

**Table 5:** The percentage of secondary structure of Hst5, P113, hybrid peptides and di-18Hc.

		Secondary structure (%)				
Peptide	Solvent	Helix	Beta	Turn	Random	Total
	10mM NaPB	0	19.3	6.2	74.5	100
Hst 5	50% TFE	76.2	7.3	16.4	0.0	100
	Laminarin	0.0	21.7	16.3	62	100
	10mM NaPB	0.0	0.0	4.7	95.3	100
P113	50% TFE	4.4	34.8	0.0	60.8	100
	Laminarin	2.8	0.2	24.7	72.2	100
	10mM NaPB	0.0	27.3	11.4	61.3	100
HHP1	50% TFE	36.8	13.5	20.0	29.7	100
	Laminarin	10.3	25.4	19.7	44.5	100
	10mM NaPB	0.0	29.2	0.0	70.8	100
di-PH2	50% TFE	10.1	32.9	5.2	51.8	100
	Laminarin	20.5	0.0	18.4	61.1	100
	10mM NaPB	0.0	22.0	14.8	63.2	100
di-WP2	50% TFE	7.8	25.7	13.9	52.5	100
	Laminarin	0.0	24.5	20.8	54.7	100
	10mM NaPB	3.8	29.2	5.5	61.5	100
di-18Hc	50% TFE	47.0	10.4	14.2	28.4	100
	Laminarin	29.2	29.7	10.8	30.3	100

### 3.6 Killing Kinetic Study

**Fig 4:**
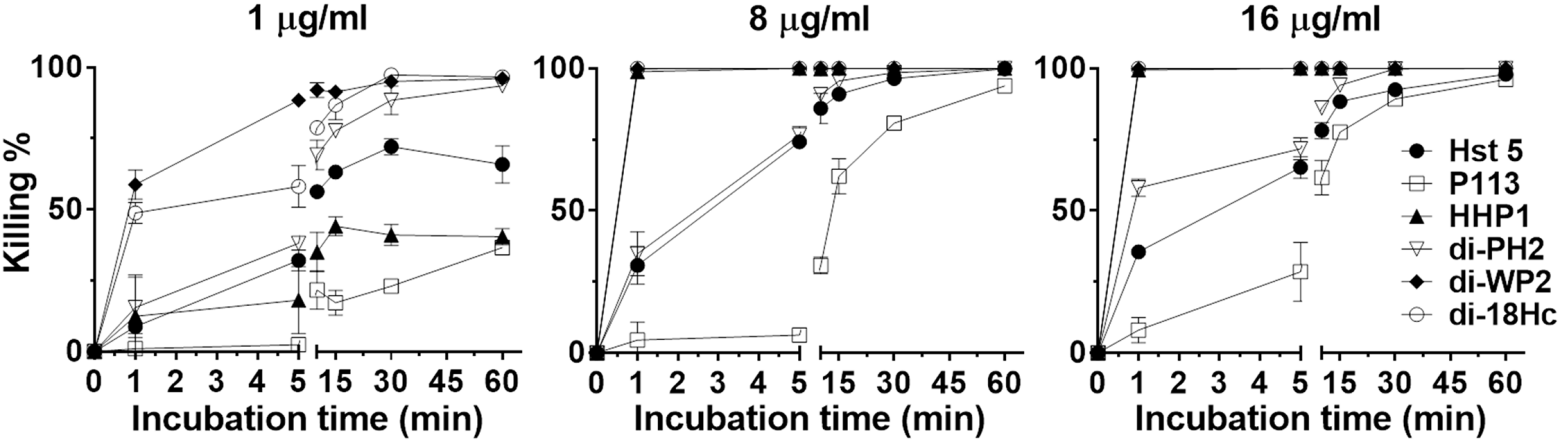
Killing kinetics of Hst5, P113, di-18Hc, hybrid peptides against *Candida albicans*. Killing kinetics of peptides against *C*. *albicans* was measured by colony count assay. Candida cells were treated with a different concentration of peptides at a determined time. 10 mM NaPB was used as a control. Killing percent was calculated with the following equation: Killing % = (number of control cell—number of treated cell)/control cell × 100.

As previously found and also observed in the current study, halocidin (di-18Hc) is a membrane-attacking peptide with fast kinetics (within 30 second). In this study, we compared the killing kinetics of hybrid congeners with halocidin, P113 and Hst 5 (Fig 4). At low concentration (1 μg/ml), all the dimeric hybrid congeners (di-PH2, di-WP2, di-18HC) exhibited relatively high killing compared to the other peptides by reducing 99% cell viability in 1 hour. However, when high concentrations were used (8 and 16 μg/ml), di-WP2, HHP1 and di-18Hc but not di-PH2 killed 99% of cells in just 1 minute. Interestingly, di-18Hc, which is known as a membrane-attacking peptide [15], killed the cells more quickly and effectively than Hst 5 (or P113), which is translocated into the cells followed by the initiation of killing activity. These results along with MIC data offer several insights into the killing mechanisms of the peptides.

First, membrane-active peptides that induce the death of pathogens by destabilizing the membrane typically operate at much faster rates (kinetics) than translocating peptides.

Second, we presumed that di-PH2 exerts its anti-candida activities after translocating into the cytosol. Although it kills 99% of the viable cells at 1 μg/ml, the relatively longer time required for the process at every concentration led us to decide that the killing mechanism for di-PH2 is similar to those of Hst 5 and P113.

Third, the killing mechanism of di-WP2 is similar to that of di-18Hc, i.e. it kills cells by attacking the membrane. With these results, we attempted to perform confocal experiments to confirm whether the hybrid peptides act on the Candida cell membrane or enter the cytoplasm for their antimicrobial activity.

### 3.7 Localization of Hst 5, P113, di-18Hc and Hybrids Peptides during *C*. *albicans* Killing

**Fig 5:**
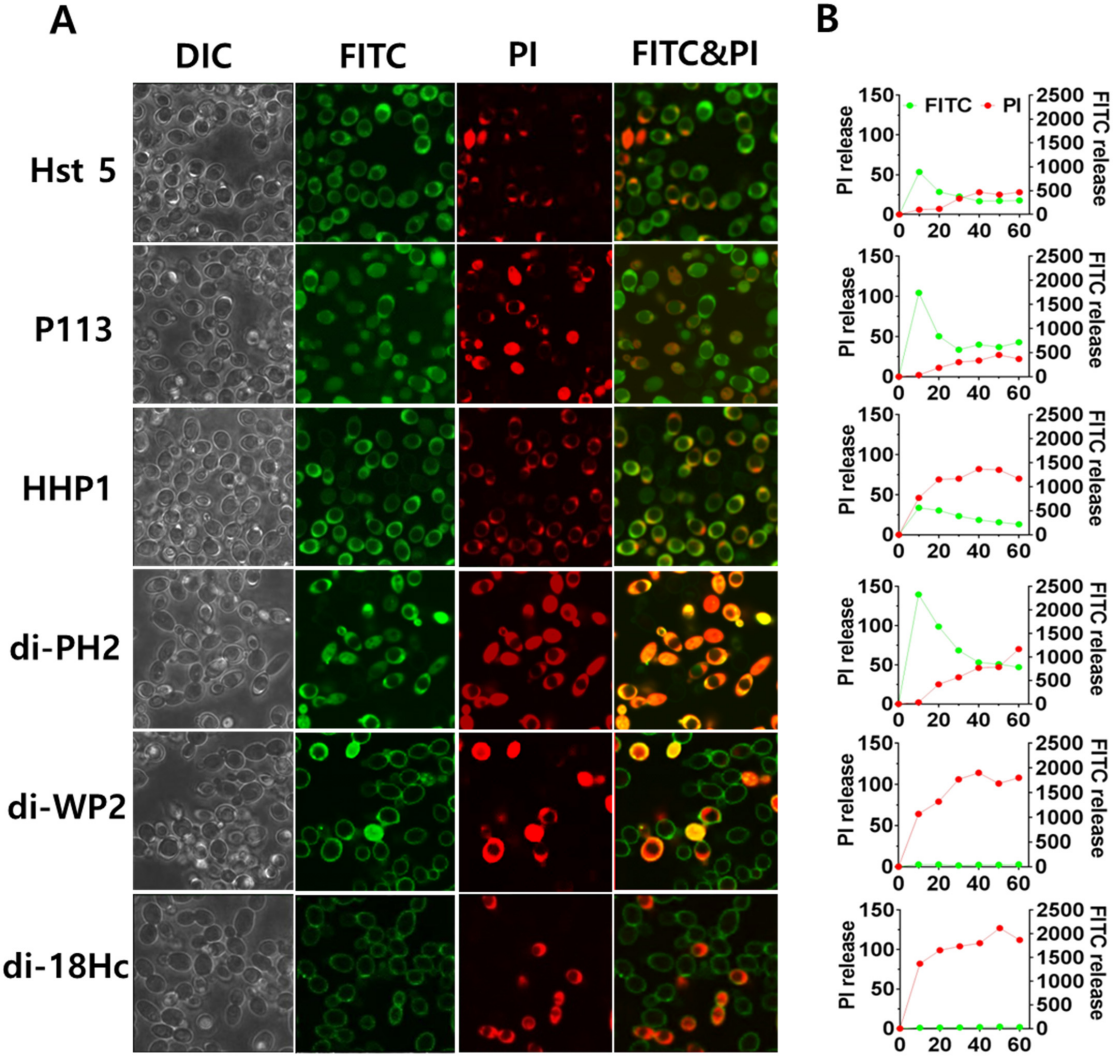
Localizations of FITC-peptides in *Candida albicans* cells. *C*. *albicans* cells were incubated for 10 minutes with 10 μg/ml of FITC-peptides and visualized by confocal microscopy. The left panel shows DIC fluorescent and FITC-PI merge images of Candida cells with FITC-peptides and PI (5 μg/ml). The right panel shows FITC-peptides and propidium iodide uptake assay. FITC-peptides and PI uptake was measured for 60 min in Perkin Elmer 2030 reader (VictorX 3).

Intracellular translocation or membrane attacking of an AMP is the two important phenomena for candidacidal targets. For example, intracellular localization is required for the toxic effects of Hst 5 and P113 [10, 11] in contrast to di-18Hc, which is reported to be a membrane attacking peptide [15]. To determine whether hybrid peptides could be translocated into cytoplasm or act on membrane for their killing activities, the cellular localization of hybrid peptides was observed by confocal fluorescence microscopy using propidium iodide (PI) and FITC-labeled peptides (10 μg/ml, F-peptides) (Fig 5A). When F-Hst5 or F-P113 was incubated with Candida cells for 10 min, F-Hst 5 or F-P113 accumulated within the cytosol followed by vacuole expansion and PI entry. Among the hybrid peptides, F-di-PH2 and F-HHP1 localized into the cytoplasm in a similar manner as Hst 5. In contrast, F-di-WP2 accumulated in the surface of the cell without translocation into the cytoplasm, followed by PI entry similar to F-di-18Hc (S1 Video). Interestingly, F-di-WP2 was observed in the cytosol of 5~10% cells, indicating that F-di-WP2 may possesses the characteristics of direct lytic action and membrane permeabilization (Fig 5A). In the FITC-peptides and propidium iodide uptake assay (Fig 5B), the FITC intensity of F-di-WP2 did not increase in Candida cells similar to F-di-18Hc, whereas the FITC intensity of Hst 5, P113, HHP1 and di-PH2 increased in a time dependent manner.

### 3.8 Candidacidal Activities of Membrane Attacking Peptides Are Not Affected by Energy Depletion

**Fig 6:**
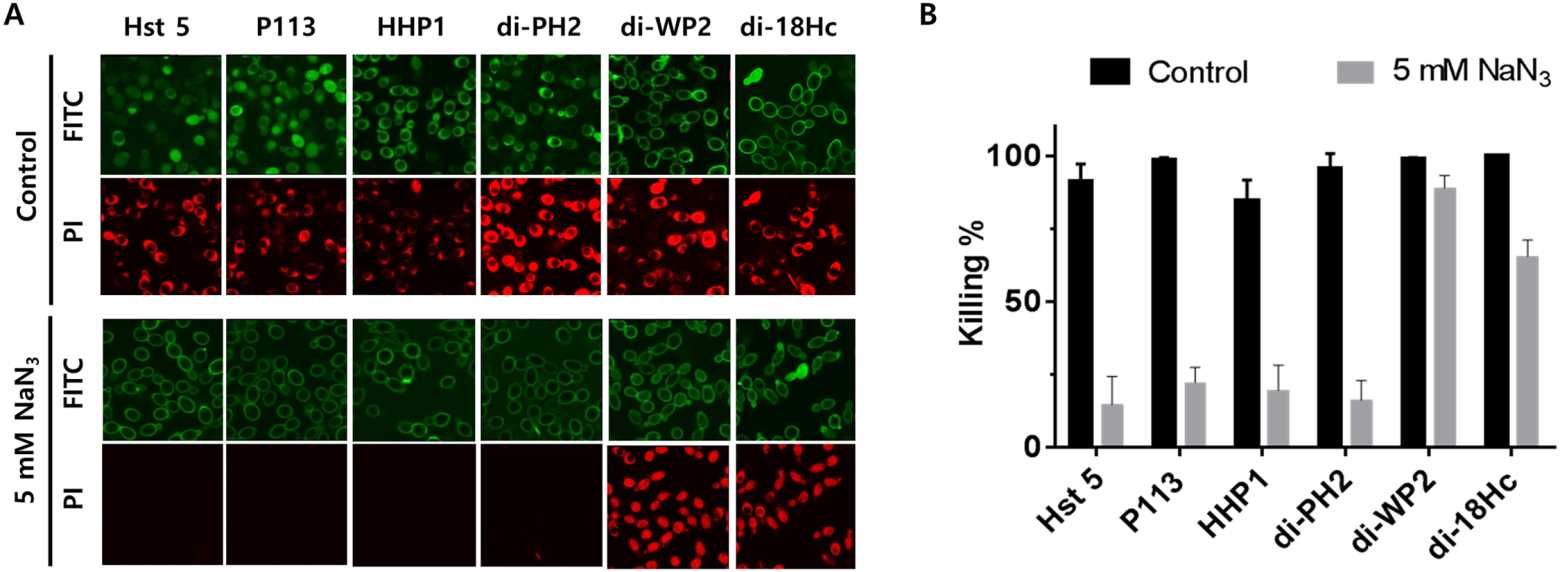
The effects of azide on killing activity of peptides. Candida cells were pre-incubated with 10 mM NaPB containing 5 mM NaN_3_ at 30°C for 60 min then washed prior to addition of F-peptides. F-Hst 5, F-P113, F-HHP1 and F-di-PH2 showed (A) reduced levels of cytosolic uptake and (B) killing activity in azide (NaN_3_, 5 mM) treated cells, whereas F-di-WP2 and F-di-18Hc showed equivalent amounts of cell wall binding to azide-treated cells and killing activity as untreated controls.

Intracellular localization or endocytotic mediated uptake of Hst 5 is energy dependent. Sodium azide is a known ATP reducer for Candida cells and it reduced intracellular transport and endocytotic mediated uptake of Hst 5 [10, 28]. To study the mechanism of hybrid peptides in more detail, the effects of azide on the candidacidal activity of the peptides were tested by confocal microscopy and colony count assays (Fig 6). In confocal microscopy, Hst 5, P113, di-PH2 and HHP1 were not translocated into cells in the presence of azide and PI influx into these peptide-treated cells was completely inhibited (Fig 6A). In contrast, di-WP2 and di-18Hc successfully bound to the cell surface and induced vacuolar expansion and PI uptake, similar to their effects in normal conditions. The antimicrobial activities of Hst 5, P113, di-PH2 and HHP1 in energy depleted cells (pre-incubated for 60 min in 5 mM NaN_3_) were significantly reduced, whereas the anti-candida activities of di-WP2 and di-18Hc were not inhibited by pre-incubation with NaN_3_ (Fig 6B). These results indicated that the candidacidal activities of di-WP2 and di-18Hc were not affected by energy depletion whereas sodium azide affected the activity of the peptides (Hst 5, P113, HHP1 and di-PH2) that could be translocated into cells.

### 3.9 ROS Production

**Fig 7:**
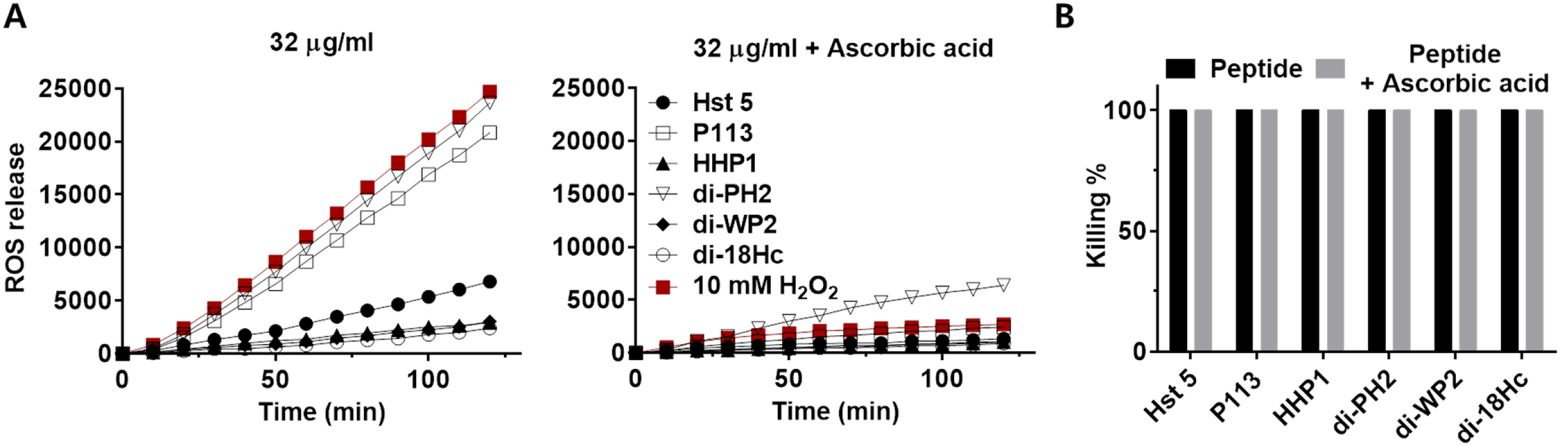
The effect of peptides on ROS generation. Time-dependency of DCF fluorescence as a measure of ROS generation (A) and killing activity (B) were determined in Candida cells treated with peptides (32 μg/ml) or peptides + Ascorbic acid (200 μg/ml) by Perkin Elmer 2030 reader (VictorX 3) and colony count assay, respectively. The fluorescence obtained with the cells treated with 10 mM of H_2_O_2_ served as positive control and the cells without peptide served as negative control. The fluorescent intensity of DCF was recorded every 10 min for 120 min at excitation and emission wavelengths of 485 and 530 nm, respectively.

Antibiotic lethality induces ROS (reactive oxygen species) generation as a consequence of downstream physiological alteration and stress response to drug mediated disruption of the target process [29]. The formation of ROS has been suggested to be one of the secondary events of fungicidal mechanisms of histatin 5 [30, 31]. In order to determine whether the killing mechanism of hybrid peptides were related with ROS production, DCFH-DA, which is oxidized by ROS in the presence of cellular peroxidases to yield 2′, 7′-dichlorofluorescein (DCF), a compound that emits a strong fluorescence, was used to monitor the generation of ROS in the Candida cells followed by incubation with the peptides at one concentrations (32 μg/ml) for 120 min. As shown in Fig 7A, a constant increase in fluorescence intensity was observed in cells treated with Hst 5, P113 and di-PH2, whereas little fluorescence was observed in cells treated with HHP1, di-WP2 and di-18Hc. The highest ROS level was observed in cells treated with di-PH2. Treatment with the antioxidant ascorbic acid decreased ROS levels in cells treated with each of the peptides. However, the decrease of ROS levels by ascorbic acid could not reduce the candidacidal activity of the test peptides in colony count assays (Fig 7B), suggesting that ROS production may not be a major event for the antimicrobial activity of the peptides. di-WP2 and di-18Hc, which appear to be membrane attacking peptides in confocal experiments, did not induce increased ROS levels in Candida cells. However, the peptides (Hst 5, P113 and di-PH2) that could be translocated into cells produced ROS in Candida cells, with the exception of HHP1. Interestingly, ROS production in the cells treated with HHP1 did not increase although HHP1 initiates its activity followed by translocation into the cell, suggesting that HHP1 might have a different intracellular killing mechanism compared to the other peptides, Hst5, P113 and di-PH2.

## 4.0 Discussion

Antimicrobial peptides (AMPs) are frequently synthesized these days by synthetic laboratories in order to achieve more biological activities. It was reported that subtle changes in amino acid sequences of short peptides might result in increased antimicrobial activity. For instance, two variants of Hst 5, dhvar 4 and dhvar 5, showed higher fungicidal kinetics than Hst 5 [12]. Dyanne Brewer and Gilles Lajoie (2002) used conformational information for Hst 3 and synthesized peptide 4 by optimally replacing cysteine residues to form a cyclic and extended loop structure [32]. Peptide 4 was found to be 100 times more potent than Hst 5 against *S*. *cerevisiae* [32]. Hybrid congeners were also synthesized previously by conjugating different amino acid sequences from two different peptides to attain higher antimicrobial activity [33, 34]. In this study, we synthesized several histatin 5 and halocidin hybrid peptides to design an antibiotic of peptide with salt resistance and low cytotoxicity. Among the hybrid peptides, di-PH2 had stronger activities than other hybrid peptides and control peptides against all tested Candida strains. The MIC values of these peptides were 1–2 μg/ml. Moreover, none of the hybrid peptides was cytotoxic in the hemolytic assay and cell-based cytotoxicity assay. In 150 mM salt condition, di-WP2 showed its killing activity against only two non-candida strains (*C*. *guilliermondi* and *C*. *tropicalis*). Although *Candida albicans* has been the major species responsible for causing candidiasis, *C*. *tropicalis*, the second most common Candida isolated [35], shows 3–66% of all Candida bloodstream isolates worldwide [36, 37] and *Candida guilliermondii*, an emerging pathogen that causes invasive infections mainly in immunocompromised and immunocompetent patients, accounts for 1% to 3% of candidemia cases [38]. Furthermore, *Candida guilliermondii* is resistant to echinocandins, the recommended first-line therapy, with MICs from 2- to 100-fold higher than those for other Candida spp [39, 40]. Because di-WP2 remained active at NaCl concentrations up to 150 mM salt condition and showed low cytotoxicity, di-WP2 might be developed as a new drug for specific targeting *C*. *guilliermondi* or *C*. *tropicalis*. Obviously, further research will be needed to clarify this interesting issue in the future.

Several mechanisms for peptide uptake followed by cytosolic translocation have been described, including vacuolar localization and expansion, spatial restriction site-induced disrupted plasma membrane, destabilization of the pore by enhanced electrostatic repulsion, and endosomal mediated or physically mediated mechanisms [10, 41–43]. It was previously shown that Hst 5 can be translocated into the cytoplasm by cellular transporters, by direct transfer across the membrane and by endocytosis [41]. In this study, Hst 5, P113, HHP1 and di-PH2 were found to be translocated to the cytosol upon cellular uptake as evidenced by the increased green fluorescence. The cellular uptake of these peptides was followed by both a ‘vacuole expansion’ mechanism and vacuole localization. On the other hand, neither intracellular uptake nor cytosol translocation was observed with di-WP2 and di-18Hc as indicated by the non-detectable FITC signal. This finding matched our conclusions on the killing kinetics. Also, a higher PI uptake rate was evidenced by the intense red signals of these two peptide congeners, indicating faster killing kinetics for di-WP2 and di-18Hc. However, surprisingly, HHP1 was found to be translocated into the cytosol even though HHP1 showed a fast killing rate. In FITC-peptides and propidium iodide uptake assay, F-HHP1 showed high signals in both the green and red channels. As PI uptake continued in the candidacidal event, the red channel signal of HHP1 was comparable to those of di-WP2 and di-18Hc, which explains why HHP1 have a fast killing rate. From this result, we assumed that HHP1 might possess a different mechanism compared to the other translocated peptides (Hst 5, P113 and di-PH2). Indeed, intracellularly translocated peptide congeners such as di-PH2 and P113 induced intense ROS generation that was higher than the amount generated by Hst 5 treatment. However, HHP1, which is a translocated peptide, did not induce a significant amount of ROS. This led us to postulate that HHP1 might possess a completely different pathway for fungicidal activity compared to Hst 5, P113 and di-PH2, even though ROS formation is a consequence event of killing mechanism of Hst 5. In addition, it is difficult to conclude that P113 and di-PH2 mediate a ROS dependent killing mechanism in *C*. *albicans* because the killing activities of these peptides were not reduced by ROS inhibitor.

The mode of action for P113 is similar to that of Hst 5 [13]. However, it was found that replacement of the lysine (Lys) residue in the second and tenth amino acid position of P113 resulted in near complete inhibition of translocation and reduced the killing of *C*. *albicans*, suggesting that the specific lysine residues at 2 and 10^st^ of P113 are important to facilitate intracellular translocation. In this study, however, HHP1 can be translocated although the specific lysine residue at 2^st^ of P113 was changed in HHP1. Furthermore, di-PH2, which has KFH sequence at C-terminal, could be translocated into the cells, whereas no translocation event was observed when candida cells were treated with F-di-PH1, which has KGVLA sequence instead of KFH sequence at C-terminal (S1 Fig). Furthermore, The MICs of F-di-PH1 against *C*. *albicans* showed 2–8 higher values compared to other peptides containing control peptides (Table 4). Indeed, di-WP2 could be also translocated into cytoplasm of 10–20% Candida cells even though di-WP2 showed the membrane attacking characteristics in confocal microscopy. This led us to believe that translocation of di-PH2 and HHP1 was encouraged somewhat by the KFH residue at the C-terminal. Although this issue warrants further research, we found at least one clue by closely examining the sequences of amino acid residues for these hybrid peptides. We suggest that KFH sequence at the C-terminal of the hybrid peptides is important to facilitate cytosolic translocation,

In conclusion, altogether, our data showed that hybrid congeners might be great alternatives to arrest and eliminate the critical limitations of existing antimicrobial peptides. Amino acid residual compartmentation and arrangement in this regard have variable effects on the activities of the congeners that should be identified and propagated pharmacologically to determine the best possible candidate for antimicrobial drug development. With its anti-candida activity at 150 mM NaCl condition and low cytotoxicity, di-WP2 deserves special attention for the development of future antibiotics against *C*. *guilliermondi* and *C*. *tropicalis*. Another hybrid congeners, di-PH2 and HHP1, should be recognized as promising due to its activities but with room for further improvements. Additionally, in ongoing attempts to understand the mechanisms of these hybrid congeners, comparative studies have revealed important phenomena on cytosolic translocation and membrane interaction. Depending on several other factors, the C-terminal amino acid residue containing KFH sequence plays a pivotal role for translocation of the hybrid peptides as well as P113 across the membrane.

## Supporting Information

S1 Fig Localizations of FITC-di-PH1 in *Candida albicans* cells. (TIF)

S1 Video Time-lapse confocal microscopy for localizations of FITC-di-WP2 and PI uptake in *Candida albicans* cells. (AVI)
