# Supplementary figures and images for: Antifungal Activity and Action Mechanism of Histatin 5-Halocidin Hybrid Peptides against Candida ssp
Source: PLoS One. 2016 Feb 26;11(2):e0150196. doi: 10.1371/journal.pone.0150196 (PMC4769088; doi:10.1371/journal.pone.0150196)

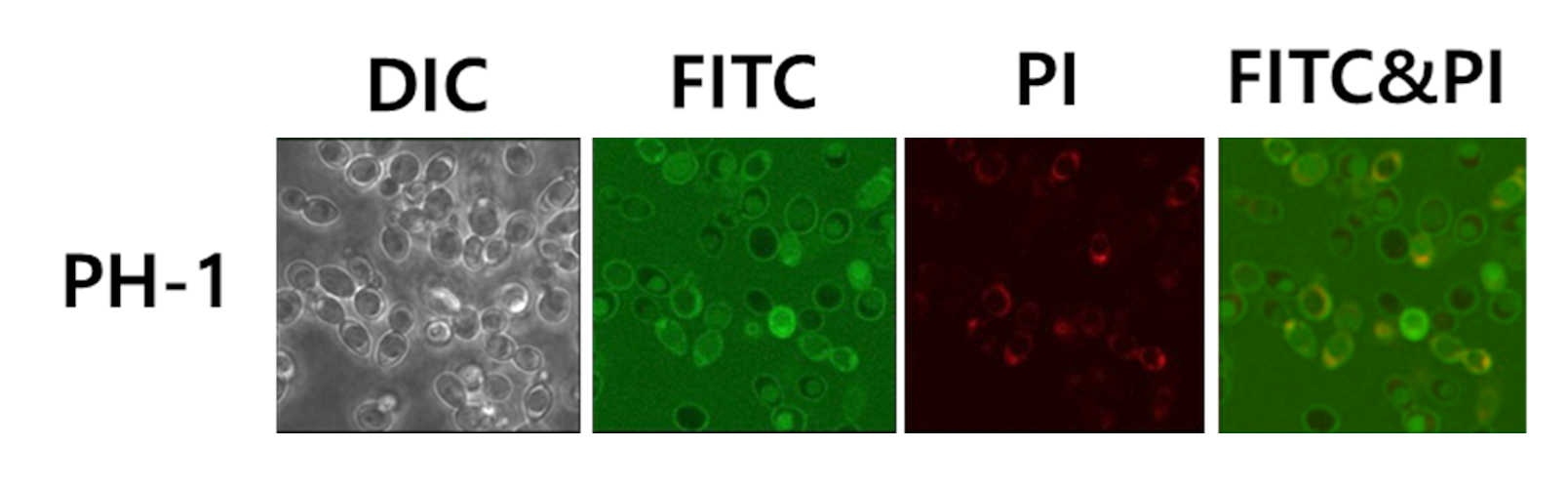

Supplement: S1 Fig — (TIF) [file pone.0150196.s001.tif]
